# The Criminal Law Amendment Bill

**Published:** 1917-05-19

**Authors:** 


### THE CRIMINAL LAW AMENDMENT BILL.

This Bill has now passed through the ordeal of the Grand Committee, and awaits the Report stage in the House of Commons. It is vitiated, as all Acts or Bills of Parliament are, by the inability of legislators to express themselves competently in the English language. Whether the incompetence is the incompetence of the Parliamentary draughtsman, or of the Home Secretary, or of the legislation conducted in Grand Committee, does not much matter, but it is a strange commentary upon the system of education under which they are all brought up that not only cannot they express themselves, but amongst the whole lot of them it appears that not one has sufficient knowledge of his own tongue to detect a patent and glaring ambiguity when it stares him in the face. Subsection (1) of section 3 imposes a penalty upon every girl who is convicted "of loitering or importuning passengers for the purposes of prostitution or solicitation, or of any offence of a like nature." We may pass the improper plural of "purposes" when qualified by "of prostitution *or* solicitation," for no legislator, no Home Secretary, and no Parliamentary draughtsman can be expected to be acquainted with the rudiments of grammar; but we cannot pass the previous portion of the clause. Can the girl be convicted of loitering *simpliciter*, or must she, before she can be convicted have been loitering for the purpose of prostitution or of solicitation? It is impossible to tell from the construction of the sentence which is meant. But the difference is vital. The *Morning Post* assumes without question or doubt that mere loitering for any purpose is enough to justify conviction, and no doubt it is open to any magistrate to take the same view. If this view is taken, it is, as the *Morning Post* points out, in the power of any policeman to arrest any girl who is looking in at a shop window or waiting on the kerb for a 'bus, and though in few such cases would there be a chance of a conviction, that is not the point. The point is the unlimited opportunity and temptation to levy blackmail that is put into the hands of the police. To such temptation no body of men ought to be subjected. It is true that this is a matter of morals rather than of medicine, but the Bill purported, when it was introduced, to be a Bill of medicine rather than of morals, though it has now changed its character; and though The Hospital is a medical and an institutional paper, men do not cease to be citizens when they take up the practice of medicine or the administration of a hospital.

The Bill has all the appearance of a Bill introduced at the instance of popular clamour, by a Government that has been told that it "must do something," and has no clear idea of what it ought to do; so it "does something" with the fervent hope that it is not doing more harm than good, and on the tacit understanding, made with itself, that it will at least allay the clamour, and thus perhaps need not be very stringently enforced. Clause 5, which makes it an offence for a person suffering from venereal disease in a communicable form to have sexual intercourse with any other person, or to solicit or invite any other person, etc., is open to manifest and horrible abuse. Obviously it might be used in the most nefarious manner for the most nefarious purposes, as a recent prosecution for blackmailing has shown. In this case the blackmailers were exposed, caught, and convicted; but would the prosecutor have had the courage to go into court if they had had this terrible weapon in their hands, and if it should have happened that a random shot had hit the bull's-eye, and that the prosecutor did happen to be suffering from venereal disease? Would he have run the fearful risk of himself appearing in the dock and being sentenced to two years' hard labour? The Bill has been called a persecuting Bill. It might fitly be called a Bill for the Promotion of the Practice of Blackmailing. We trust this aspect of it will receive the attention of the House of Commons during the consideration of the Bill as reported.

